# Chemical Profile and Antioxidant, Anti-Inflammatory, Antimutagenic and Antimicrobial Activities of Geopropolis from the Stingless Bee *Melipona orbignyi*

**DOI:** 10.3390/ijms18050953

**Published:** 2017-05-03

**Authors:** Helder Freitas dos Santos, Jaqueline Ferreira Campos, Cintia Miranda dos Santos, José Benedito Perrella Balestieri, Denise Brentan Silva, Carlos Alexandre Carollo, Kely de Picoli Souza, Leticia Miranda Estevinho, Edson Lucas dos Santos

**Affiliations:** 1 Research group on Biotechnology and Bioprospecting Applied to Metabolism (GEBBAM), Federal University of Grande Dourados, Rodovia Dourados Itahum, Km 12, 79804-970 Dourados, MS, Brazil; helderspk@gmail.com (H.F.d.S.); jcampos_bio@yahoo.com.br (J.F.C.); cinty.santos94@gmail.com (C.M.d.S.); josebalestieri@ufgd.edu.br (J.B.P.B.); kelypicoli@gmail.com (K.d.P.S.); 2 Laboratory of Natural Products and Mass Spectrometry, Federal University of Mato Grosso do Sul, Cidade Universitária, 79070-900 Campo Grande, MS, Brazil; denisebrentan@gmail.com (D.B.S); carloscarollo@gmail.com (C.A.C.); 3 Polytechnic Institute of Bragança, Agricultural College of Bragança, Campus Santa Apolónia, E 5301-855 Bragança, Portugal; leticia@ipb.pt; 4 Centre of Molecular and Environmental Biology, Biology Department, Minho University, Campus de Gualtar, 4710-057 Braga, Portugal

**Keywords:** HPLC-DAD-MS 1, phenolic compounds 2, lipid peroxidation 3, hyaluronidase 4, mutation 5, microorganisms 6

## Abstract

Geopropolis is a resin mixed with mud, produced only by stingless bees. Despite being popularly known for its medicinal properties, few scientific studies have proven its biological activities. In this context, the objective of this study was to determine the chemical composition and antioxidant, anti-inflammatory, antimutagenic and antimicrobial activities of the *Melipona orbignyi* geopropolis. The hydroalcoholic extract of geopropolis (HEGP) was prepared and its chemical composition determined by high performance liquid chromatography coupled to diode array detector and mass spectrometry (HPLC-DAD-MS). The antioxidant activity was determined by the capture of free radicals and inhibition of lipid peroxidation in human erythrocytes. The anti-inflammatory activity was evaluated by the inhibition of the hyaluronidase enzyme and the antimutagenic action was investigated in *Saccharomyces cerevisiae* colonies. The antimicrobial activities were determined against bacteria and yeasts, isolated from reference strains and hospital origin. The chemical composition of HEGP included flavonoids, derivatives of glycosylated phenolic acids and terpenoids. HEGP showed high antioxidant activity, it inhibited the activity of the inflammatory enzyme hyaluronidase and reduced the mutagenic effects in *S. cerevisiae*. In relation to the antimicrobial activity, it promoted the death of all microorganisms evaluated. In conclusion, this study reveals for the first time the chemical composition of the HEGP of *M. orbignyi* and demonstrates its pharmacological properties.

## 1. Introduction

Geopropolis is an apicultural product used in popular medicine for the treatment of digestive, respiratory, visual, female fertility, and dermatosis problems, in addition to being antiseptic and immunostimulatory [1,2]. In Brazil, it is used by indigenous communities in the Amazon region [3] and in the northeast of the country [4].

This material is exclusively produced by stingless bees (Hymenoptera, Apidae, and Meliponinae) [5,6,7] found in the Neotropical region [8]. To produce geopropolis, bees add their mandibular secretions and waxes to the exudates collected from plant materials and mix mud into the result, conferring unique characteristics to this product [7]. This material is used in the beehive to seal cracks, provide mechanical protection and prevent excessive entry of air [9].

In recent years, some scientific reports have described the therapeutic properties of the geopropolis produced by different species of stingless bees, such as antimicrobial [5], anticancer [2], antioxidant [7,10], anti-inflammatory [6,11], gastroprotective [12] and antiviral [13] activities.

Regarding its chemical composition, the presence of phenylpropanoids, flavonoids [10], phenolic acids, hydrolysable tannins [7], triterpenes, saponins [14] and alkaloids [13] have been described, compounds that may be related to the biological properties of this bee product. Despite the growing interest in the pharmacological properties of the geopropolis produced by the *Melipona*, the data in the literature remain very scarce compared to those on the propolis of the *Apis mellifera* species. In the Chemical Abstract database, only 35 studies involving geopropolis were found, while more than 4000 papers studied the chemical composition and biological properties of *Apis* propolis [15].

*Melipona orbignyi* is among the species of bees that produce the geopropolis (Guérin, 1844), popularly known in Brazil as Manduri-de-Mato-Grosso [9]. This species is found in South America, specifically in Argentina, Bolivia, Paraguay and Brazil, in the latter being restricted to the states of Mato Grosso and Mato Grosso do Sul [8]. In addition to geopropolis, other bee products are also produced by this species, such as honey and propolis. Recently, Campos et al. [16] described the chemical composition and biological activities of the propolis produced by *M. orbignyi*, in the only study thus far reported on the products generated by this species.

In this context, the objective of this study was to investigate the chemical composition and antioxidant, anti-inflammatory, antimutagenic and antimicrobial properties of the geopropolis produced by *M. orbignyi*.

## 2. Results

### 2.1. Chemical Analysis

#### 2.1.1. Determination of Phenolic Compounds and Flavonoids

The content of phenolic compounds present in the *M. orbignyi* geopropolis extract was 121 ± 0.6 mg expressed in mg of gallic acid equivalents (GAE)/g of hydroalcoholic extract of geopropolis (HEGP), and the total flavonoid content was 19.9 ± 1.1 mg expressed in mg of quercetin equivalent (QE)/g HEGP.

#### 2.1.2. Chemical Composition of HEGP by High Performance Liquid Chromatography Coupled to Diode Array Detector and Mass Spectrometry (HPLC-DAD-MS)

**Figure 1:**
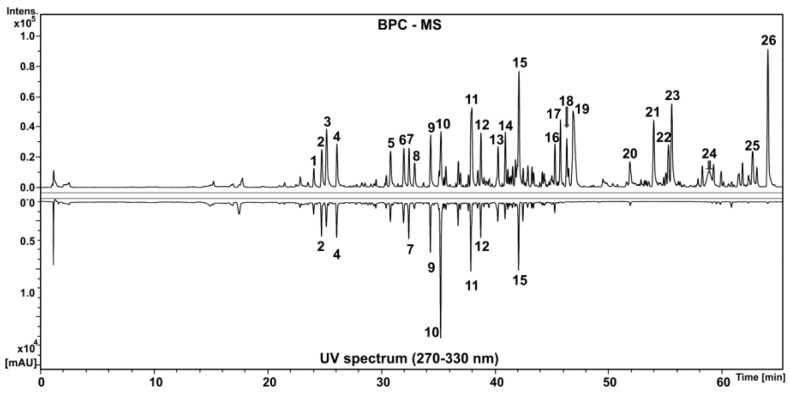
Base peak chromatogram in negative ion mode and UV spectrum in the range of 270–300 nm of the *Melipona orbignyi* geopropolis extract. Peaks 1 to 26 are identified in Table 1.

A complex composition of HEGP was observed by HPLC-DAD-MS analyses (Figure 1). Several compounds exhibited UV absorption (Table 1, compounds 1–16) and thus conjugated chromophores, including flavanones, flavanonols and glycosylated phenolic acid derivatives. The identification of substances **3**, **8**, **10** and **13** was based on their UV spectra, which exhibited an absorption band centered at 290 nm, compatible with flavanones and flavanonols [17]. The molecular formulas were confirmed from deprotonated ions with accurate mass (Table 1). The structural characterizations were also based on fragment ions yielded from B-ring cleavages and the loss of CO, as well as the losses of radical methyl (15 *u*) for methylated compounds (Table 1). These fragmentation pathways are characteristic of flavonoids [18,19]. Thus, compounds **3**, **8**, **10** and **13** were identified as aromadendrin, naringenin, methyl aromadendrin and methyl naringenin, respectively. Flavonoids **3** and **8** were already identified from the geopropolis of *Melipona interrupta* and *Melipona seminigra* [20,21].

The glycosylated phenolic acid derivatives consisted of galloyl, cinnamoyl and coumaroyl substituents exhibiting two or three *O*-phenolic acids in the structures. The peaks of **1**, **2**, **4**, **5**, **6**, **9** and **12** showed a fragment ion at *m*/*z* 169 (C_7_H_5_O_5_)^−^ in negative ion mode, confirming the presence of galloyl in the structures. In addition, compounds **4**, **6**, **9** and **12** also showed product ions at *m*/*z* 313 (C_13_H_13_O_9_)^−^ and 163 (C_9_H_7_O_3_)^−^ related to the ions [M-H-coumaroyl-H_2_O]^−^ and [M-H-galloyl-hexosyl]^−^, respectively. All this spectral information taken together allowed the putative identification of the compounds as *O*-coumaroyl *O*-galloyl-hexoside (**1**), *O*-coumaroyl *O*-galloyl-hexoside (**2**), di-*O*-galloyl *O*-coumaroyl-hexoside (**4**), *O*-cinnamoyl *O*-galloyl-hexoside (**5**), di-*O*-galloyl *O*-cinnamoyl-hexoside (**6**), di-*O*-coumaroyl-hexoside (**7**), di-*O*-coumaroyl *O*-galloyl-hexoside (**9**), *O*-cinnamoyl *O*-coumaroyl-hexoside (**10**) and *O*-cinnamoyl *O*-coumaroyl *O*-galloyl-hexoside (**12**). All the data were compatible with data previously published for these compounds [22].

The compounds at the end of the chromatogram did not absorb in the UV region and had a molecular constitution typical of terpene derivatives; they belong to the classes of sesquiterpenes (compound **19**), diterpenes (compounds **17**, **18** and **20**) and triterpenes (**21**, **22**, **23** and **25**), which are common in propolis and geopropolis [16,23,24,25]. Due to the huge variety in the skeleton arrangement of these classes and the lack of systematic information about fragmentation by ESI in the literature, these compounds were not completely identified.

### 2.2. Antioxidant Activity

#### 2.2.1. Capture of Free Radicals DPPH^•^ and ABTS^•+^

HEGP inhibited 50% of the free radicals (IC_50_) at a concentration approximately six times higher than the ascorbic acid control, in both the DPPH^•^ radical capture assay and the ABTS^•+^. In turn, it presented results similar to the reference antioxidant BHT in both trials (Table 2).

#### 2.2.2. Hemolytic Activity and Inhibition of Oxidative Hemolysis

**Figure 2:**
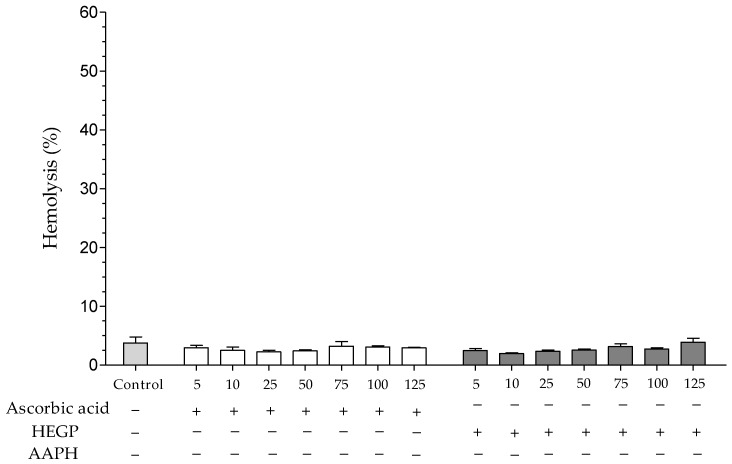
Hemolytic activity of ascorbic acid and HEGP (5–125 μg/mL), incubated with erythrocytes and 0.9% NaCl solution for 240 min, without the presence of AAPH. The control consists of erythrocytes incubated only with 0.9% NaCl solution. −: absence; +: presence.

In this assay, the hemolytic activity of HEGP and its ability to protect erythrocytes against hemolysis induced by the oxidizing agent 2,2′-azobis (2-methylpropionamidine) dihydrochloride (AAPH) were evaluated. When erythrocytes were incubated only with HEGP, in the absence of AAPH, no hemolysis was observed throughout the experimental period at the concentrations evaluated (Figure 2).

**Figure 3:**
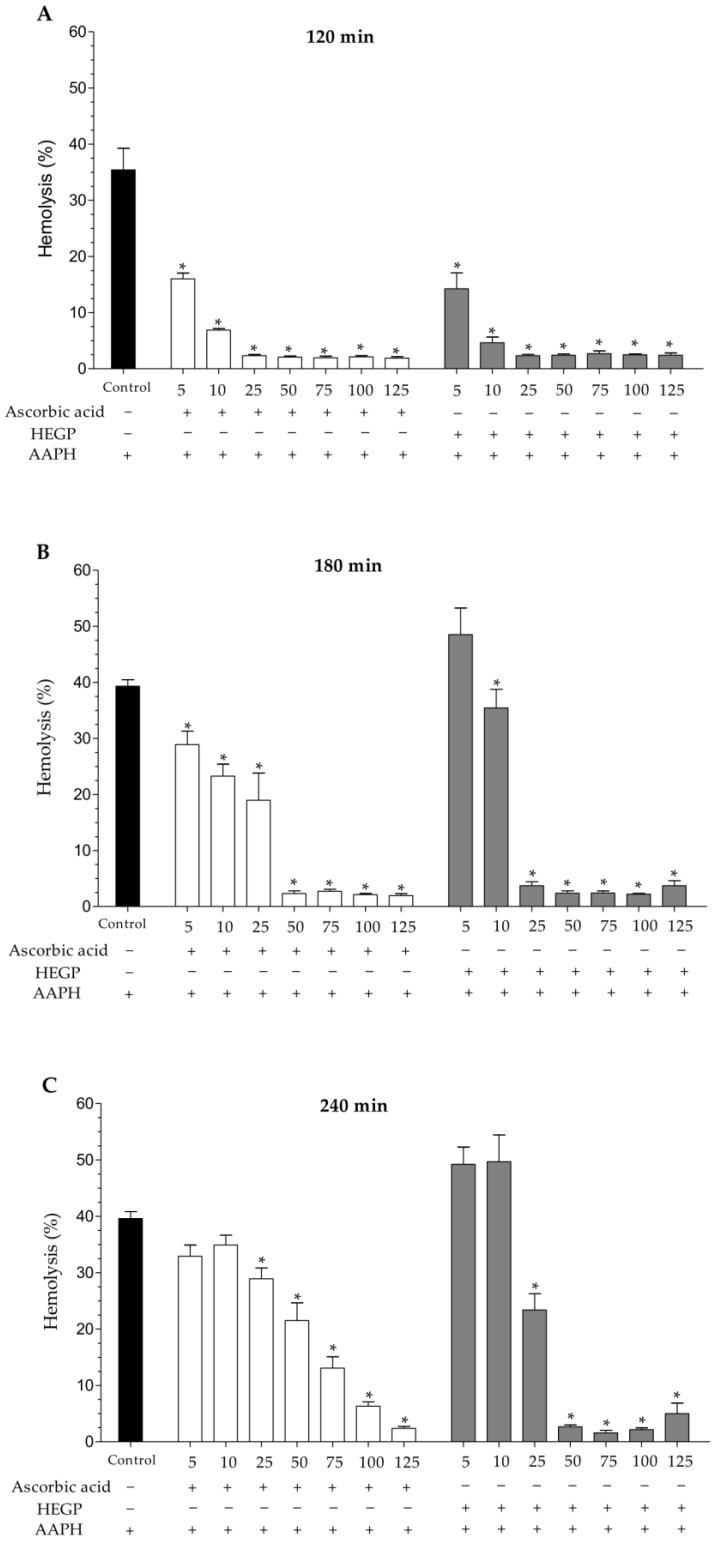
Anti-hemolytic activity of ascorbic acid and HEGP (5–125 μg/mL) incubated with erythrocytes and the oxidizing agent AAPH at: (**A**) 120 min; (**B**) 180 min; and (**C**) 240 min. The control consists of erythrocytes incubated only with AAPH solution (50 mM). * *p* < 0.05 when the treated groups were compared to the AAPH control group. −: absence; +: presence.

In evaluating the extract’s ability to protect erythrocytes against AAPH-induced hemolysis, HEGP was able to inhibit oxidative hemolysis for up to 240 min of incubation (Figure 3A–C). At concentrations of 25 and 50 μg/mL, the extract reduced hemolysis by 40.9% ± 8.0% and 93.2% ± 0.8%, respectively, after 240 min of incubation compared to the control incubated with the AAPH oxidizing agent alone. The HEGP presented higher anti-hemolytic action than the ascorbic acid control, which reduced hemolysis by 26.7% ± 4.9% (25 μg/mL) and 45.7% ± 8.0% (50 μg/mL) (Figure 3A–C).

#### 2.2.3. Dosage of Malondialdehyde

**Figure 4:**
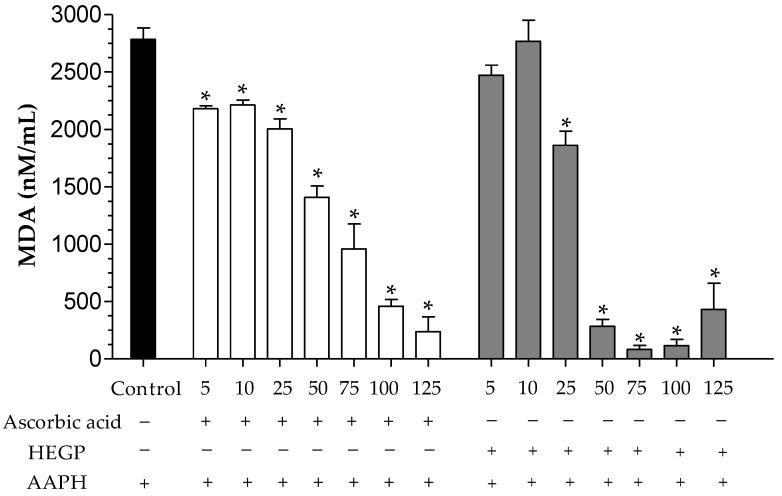
Concentration of malondialdehyde (nM/mL) in erythrocytes incubated with ascorbic acid and HEGP (5–125 μg/mL) for 240 min. The control consists of erythrocytes incubated only with AAPH solution (50 mM). * *p* < 0.05 when the treated groups were compared to the AAPH control group. −: absence; +: presence.

HEGP demonstrated the concentration-dependent reduction of MDA levels and presented better activity than the ascorbic acid control (Figure 4). At the concentration of 50 μg/mL, the extract reduced MDA levels by 89.75% ± 2.1%, while the ascorbic acid control presented a reduction of only 49.45% ± 3.5% under the same conditions.

### 2.3. Anti-Inflammatory Activity

**Figure 5:**
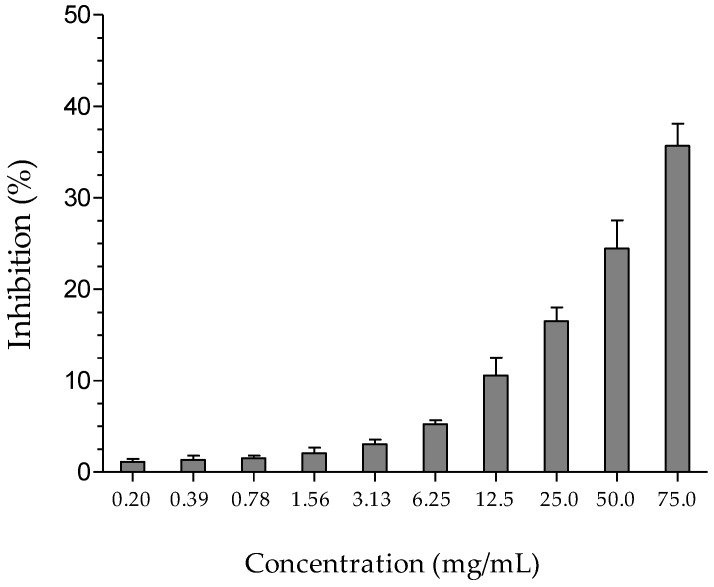
Percentage inhibition of hyaluronidase enzyme activity by HEGP (0.2–75 mg/mL).

The anti-inflammatory property of HEGP was assessed indirectly by its inhibition of the activity of the hyaluronidase enzyme. HEGP exhibited a concentration-dependent inhibition of enzyme activity, demonstrating inhibition of 35.6% ± 2.4% at the concentration of 75 mg/mL (Figure 5).

### 2.4. Antimutagenic Activity

HEGP was able to reduce the survival of *S. cerevisiae* D7 (ATCC 201137) by 48.1% ± 1.1% and 60.3% ± 3.9% at concentrations of 1.5 and 3.0 mg/mL, respectively (Table 3). When yeasts were incubated only with the mutagen EMS, there was an increase in the conversion of genes and in the quantity of mutant colonies. When incubated with HEGP in the presence of EMS, 14.6% ± 5.4% (1.5 mg/mL) and 27.5% ± 2.5% (3.0 mg/mL) reductions of gene conversion were observed. In addition, HEGP was able to reduce the amount of mutant colonies by 81.1% ± 1.1% (1.5 mg/mL) and 86.3% ± 0.4% (3.0 mg/mL) (Table 3).

### 2.5. Antimicrobial Activity

HEGP demonstrated antimicrobial activity against all evaluated microorganisms. Gram-positive bacteria were more sensitive to the action of the extract than gram-negative bacteria. The inhibition observed against the evaluated microorganisms followed the sequence: *S. aureus* > *E. faecalis* > *E. coli* > *P. aeruginosa* > *C. neoformans* > *C. albicans*. HEGP also showed action against all microorganisms resistant to antimicrobial drugs, at concentrations similar to the reference strains. In addition to inhibitory activity, HEGP showed bactericidal and fungicidal activity against all microorganisms evaluated in this study, ranging from 8.50 ± 0.28 mg/mL for *S. aureus* ATCC 6538™ to 36.1 ± 0.50 mg/mL for amphotericin B-resistant *C. albicans* ESA 97 from biological fluid (Table 4).

## 3. Discussion

Geopropolis is a beehive material of complex composition, produced specifically by species of stingless bees of the Neotropical region and popularly known for its therapeutic properties. However, despite the great diversity of species of bees capable of generating this product, few studies have investigated and described its chemical and pharmacological properties.

In this study, when investigating the chemical composition of *M. orbignyi* HEGP, different compounds were identified from the ones found in geopropolis produced by other species of stingless bees, such as derivatives of glycosylated phenolic acids. The chemical composition of geopropolis from the *M. fasciculata* and *M. scutellaris* bees presented great differences from the composition observed in this work for the *M. orbignyi* species, including benzophenones, hydrolyzable tannins (gallotannins and ellagitannins) and gallic and ellagic acid substances [7,26]. These differences in chemical composition suggest that *M. orbignyi* uses different plant sources of raw material to produce its geopropolis and/or that the production process significantly changes the composition of this material, as the genetic variability of bee species may influence this chemical composition [21]. Thus, these bioproducts from these stingless bees are unique, increasing the possibilities of identifying new bioactive compounds with pharmacological properties.

The total phenol content observed for HEGP was similar to the value obtained for the geopropolis of *M. scutellaris* (127 ± 1.9 mg GAE/g of extract) but higher than that for *M. fasciculata* (47.78 ± 0.04 mg GAE/g of extract) and that for *M. interrupt* and *M. seminigra* (419 to 4378 μg GAE/g of extract) [7,11,21]. HEGP demonstrated potent antioxidant activity, being able to inhibit the free radicals DPPH^•^ and ABTS^•+^. The ethanolic extracts from the geopropolis of *M. interrupt* and *M. seminigra* exhibited almost 40 and 30 times higher total phenol content than those from HEGP (121 ± 0.6 mg GAE/g HEGP), but the antioxidant activities of DPPH^•^ were 10 ± 0.5 and 26.3 ± 3.9 μg/mL, respectively [21]. This result demonstrates the great antioxidant potency of the compounds of HEGP, even though it had a lower total phenol content, its antioxidant activity in DPPH^•^ was 18.3 ± 2.8 μg/mL. In addition, as the chemical compositions of these geopropolis types and HEGP were different, especially with respect to the glycosylated derivatives of phenolic acids, this result demonstrates the importance of the structural characteristics of the constituents for this activity. The phenolic acids and flavonoids have hydroxyl groups in their rings [7], conferring the ability to stabilize unpaired electrons, which are typical of free radicals, thus acting as antioxidants [27].

In addition, HEGP presented in vitro antioxidant action in a human biological model by inhibiting lipid peroxidation in erythrocytes induced by an oxidizing agent. The extract showed better results than the standard antioxidant ascorbic acid, in terms of both anti-hemolytic action and the reduction of MDA levels, exhibiting a greater protective effect. This result can be attributed to the synergistic action of its compounds, including the different types of phenolic constituents, which offer excellent cellular protection against lipid peroxidation, acting as hydrogen donor agents, singlet oxygen reducers and superoxide radicals, antioxidant enzyme activators and metal chelators [27,28]. Some authors classify glycosylated derivatives of phenolic acids as a new type of hydrolysable tannin, similar to the ones described in plants such as *Balanophora harlandii* (Balanophoraceae) [29]. Among the phenolic compounds identified in the HEGP, tannins are described as acting as metal ion chelators [30] and have many hydroxyl groups in their phenolic rings, promoting the antioxidant activity of these compounds [31].

In addition, the terpenes constitute another class of compounds that display important antioxidant action. Triterpenes, one of the terpene subtypes identified in HEGP, are described as acting to capture free radicals, including superoxide anion and hydroxyl radical, and acting to protect against the lipid peroxidation process [32]. In addition, they are capable of modulating the activity of antioxidant enzymes, such as superoxide dismutase, catalase and glutathione peroxidase [33]. Mechanisms similar to the ones described for sesquiterpenes [34], compounds also identified in HEGP.

The excess of free radicals in the body, without an efficient system to neutralize antioxidant agents, triggers the process of oxidative stress, which is responsible for damage to various cellular biomolecules, such as nucleic acids, proteins and DNA [35]. This process is often related to the development of neurodegenerative diseases such as Alzheimer’s and Parkinson’s, as well as cancer, diabetes, atherosclerosis [36] and chronic inflammation [37].

Inflammation is due to the action of several mediators that promote vascular and cellular events [38]. Inflammatory cells produce mediators, such as arachidonic acid metabolites, cytokines and chemokines, which aid in the production of reactive species [37]. Under normal physiological conditions, these oxidative and immunological processes aid in the response against stressors and pathogens [39]. However, the constant activation of these processes may lead to an increased risk of more serious diseases [37,39], such as diabetes [40] and obesity [41].

Natural products have been the objects of studies for the development of new anti-inflammatory drugs [6]. Among them, geopropolis has presented satisfactory results, such as the geopropolis extract of *Melipona scutellaris*, which promoted anti-inflammatory action through the inhibition of IL-1B and TNF-α [11] and decreased neutrophil migration in vivo [6]. In this study, HEGP demonstrated anti-inflammatory activity by inhibiting the enzyme hyaluronidase, reducing the degradation of hyaluronic acid. This polysaccharide performs important biological functions in the organism, such as cell proliferation, differentiation and tissue repair [42]. According to Pascoal et al. [43], the degradation of hyaluronic acid by the enzyme hyaluronidase can lead to bone loss, pain and inflammation.

Sesquiterpenes are among the compounds with anti-inflammatory potential reported in HEGP; they inhibit the pro-inflammatory transcription factors NF-κB and STAT3 [44]. In addition, phenolic compounds are described as anti-inflammatory agents because they modulate the expression of cytokines [45] and pro-inflammatory enzymes [46], such as nitric oxide synthase and cyclooxygenase-2 [47]. Of the phenolic compounds present in HEGP, aromadendrin exerts anti-inflammatory action by reducing the release of inflammatory factors MCP-1 and IL-8 in normal keratinocytes stimulated with interferon and histamine [48]. In addition, methyl aromadendrin (4′-methyl ether), a compound also present in HEGP and commonly found in green propolis produced by *Apis mellifera*, can reduce reactive species produced by neutrophil metabolism during the inflammatory process [49].

The reactive species generated during the process of chronic inflammation may contribute to the onset of mutagenesis and carcinogenesis [50]. In this way, the identification of compounds with antioxidant and antimutagenic properties may have great therapeutic importance. Substances with such activities have been found in many natural products, such as plants [51] and bee products [42].

In addition to antioxidant properties, *M. orbignyi* HEGP demonstrated antimutagenic potential. In this study, the extract decreased the survival of yeast *S. cerevisiae*, which may be related to its fungicidal action. However, it was able to reduce the frequency of gene conversion and the amount of mutant colonies induced by the mutagenic agent EMS. Some studies suggest that the antimutagenic activity of *Apis mellifera* propolis is related to its antioxidant activity [52]. Phenolic compounds and terpenes are well known for their antioxidant properties as well as for acting as antimutagenic agents. In addition, their intake is related to the reduction of cancer risks [53]. Diterpenes are described as being capable of repairing DNA errors, also related to their anticancer activity [54].

Another biological activity presented by HEGP was antimicrobial action, including effectiveness against hospital strains resistant to antimicrobial drugs, such as methicillin-resistant *Staphylococcus aureus* gram-positive bacteria, cephalosporin-resistant *Escherichia coli* gram-negative bacteria, and amphotericin B-resistant *Candida albicans* yeast. Currently, the intensive use of antimicrobial drugs, both for therapeutic and prophylactic purposes, has resulted in the development of multiresistant strains, becoming a worldwide problem [55].

Antimicrobial resistance is a worrisome public health issue, especially in hospital environments [56]. The efficacy of antibiotics in the treatment of common infections has decreased in recent years [57], resulting in outbreaks of multiresistant strains [58]. Natural products are among the alternative agents against microbial action, considering the synergistic effects of their compounds [55].

Among the natural substances that present antimicrobial action, phenolic compounds act through the permeabilization of the microbial cytoplasmic membrane, inhibition of the synthesis of nucleic acids of gram-negative and gram-positive bacteria [59], inhibition of the synthesis of ATP and interruption of electron transport [60]. In addition to these mechanisms, flavonoids form complexes with proteins through hydrogen bonds, and their antimicrobial activity is related to the inhibition of microbial adhesins, enzymes and protein transport [28].

Among the flavonoids present in HEGP, flavanone naringenin promotes the inhibition of bacterial cytoplasmic membrane function, reducing its motility [59], even against methicillin-resistant *Staphylococcus aureus* [28]. In addition, glycosylated phenolic derivatives are present in the extract and are described by some authors as new types of hydrolysable tannins, which exert antimicrobial action by breaking the permeability of the membrane of different microbial strains [61].

## 4. Materials and Methods

### 4.1. Preparation of the Hydroethanolic Extract of Geopropolis

Samples of geopropolis were obtained in Mato Grosso do Sul, Brazil (22°13’12’’ S–54°49’2’’ W). The hydroethanolic extract of geopropolis (HEGP) was prepared in the proportions of 240 mL of 70% ethanol for each 80 g of powder geopropolis. The solution was then kept shaking in a closed container for 24 h at room temperature. Subsequently, the extract was filtered, concentrated in a rotary evaporator (Gehaka, São Paulo, SP, Brazil) at 40 °C and lyophilized to obtain the dry extract. The yield of HEGP was 3.49%, and the final freeze-dried was stored at −20 °C protected from light.

### 4.2. Determination of Phenolic Compounds and Flavonoids

#### 4.2.1. Phenolic Compounds

The concentration of phenolic compounds present in HEGP was determined by the Folin–Ciocalteu colorimetric method. For this purpose, 0.5 mL of HEGP (100 μg/mL) was added to 2.5 mL of the Folin–Ciocalteau reagent and 2.0 mL of 14% sodium carbonate (Na_2_CO_3_) solution. Absorbance reading was performed at 760 nm after 2 h of incubation at room temperature in the dark. To produce the calibration curve, gallic acid (0.4–16 μg/mL) was used as a standard. The average of three readings was used to determine the content of phenolic compounds expressed in mg of GAE/g of HEGP.

#### 4.2.2. Total Flavonoids

To verify the concentration of flavonoids present in the extract, 0.5 mL of HEGP (100 μg/mL) was mixed with 4.5 mL of methanolic solution of aluminum chloride hexahydrate 2% (AlCl_3_·6H_2_O) and incubated for 30 min at room temperature in the dark. After this period, absorbance reading was performed at 415 nm. To produce the calibration curve, quercetin (0.4–16 μg/mL) was used as a standard. The average of three readings was used to determine the flavonoid content expressed in mg of QE/g of HEGP.

### 4.3. HPLC-DAD-MS Analyses

The HEGP (1 mg/mL) was injected into an ultra-fast liquid chromatograph UFLC (LC-20AD, Shimadzu) coupled to a diode array detector operating at 240–800 nm and a mass spectrometer with electrospray ionization (ESI) and the analyzers quadrupole–Time-of-Flight (QTOF) (micrOTOF-Q III, Bruker Daltonics) monitoring between *m*/*z* 120 and 1200 in negative and positive ion mode, equipped with a C-18 column (Kinetex, 150 × 2.2 mm id, 2.6 µm) and using an oven temperature of 50 °C. The mobile phase was deionized water (A) and acetonitrile (B), both with 0.1% formic acid (*v*/*v*), under the following gradient profile: 0–8 min 3% B, 8–30 min 3–25% B, and 30–60 min 25–80% B, followed by washing and reconditioning of the column (8 min). The flow rate was 0.3 mL/min, and the injection volume was 1 μL.

### 4.4. Antioxidant Activities

#### 4.4.1. DPPH^•^ Free Radical Capture Activity

For this experiment, 200 μL of the HEGP was mixed with 1800 μL of 0.11 mM 2,2-diphenyl-1-picrylhydrazyl (DPPH^•^) radical solution in 80% ethanol. The final concentrations of the extract ranged from 0.1 to 200 μg/mL. The mixture was incubated at room temperature in the dark for 30 min. The absorbance was measured at 517 nm in a T 70 UV/VIS spectrophotometer (PG Instruments Limited, Leicestershire, UK). Ascorbic acid and butylated hydroxytoluene (BHT) were used as reference antioxidants. As control, 200 μL of the solvent (80% ethanol) was incubated with 1800 μL of the DPPH^•^ solution. Three independent experiments were performed in triplicate. The percent inhibition was calculated from the control using the following equation:

Capture activity of DPPH^•^ (%) = (1 − Abs_sample_/Abs_control_) × 100 (1)

#### 4.4.2. ABTS^•+^ Free Radical Capture Activity

The 2,2′-azino-bis(3-ethylbenzothiazoline-6-sulphonic acid) (ABTS^•+^) radical was prepared from a mixture of 5 mL of ABTS (7 mM) and 88 μL of potassium persulfate (140 mM) and incubated for 12–16 h at room temperature in the dark. After this period, the ABTS^•+^ radical solution was diluted in absolute ethanol until an absorbance of 0.70 ± 0.05 at 734 nm was obtained in a T 70 UV/VIS spectrophotometer (PG Instruments Limited, Leicestershire, UK). Then, 20 μL of HEGP was added to 1980 μL of the ABTS^•+^ radical. The final concentrations of the extract ranged from 0.1 to 200 μg/mL. The mixture was incubated at room temperature in the dark for 6 min. The absorbance was evaluated at 734 nm in a T 70 UV/VIS spectrophotometer (PG Instruments Limited, Leicestershire, UK). Ascorbic acid and BHT were used as positive controls. As control, 20 μL of the solvent (80% ethanol) was incubated with 1980 μL of the ABTS^•+^ radical. Two independent experiments were performed in triplicate. The percentage of ABTS^•+^ radical inhibition was calculated according to the following equation:

Inhibition of the radical ABTS^•+^ (%) = ((Abs_control_ − Abs_sample_)/Abs_control_) × 100 (2)

#### 4.4.3. Antioxidant Assays in Human Erythrocytes

##### Preparation of the Erythrocyte Suspensions

Human erythrocyte assays were performed after receiving approval from the Research Ethics Committee (Comitê de Ética em Pesquisa, CEP) of the University Center of the Grande Dourados (Centro Universitário da Grande Dourados, UNIGRAN), Brazil (CEP process number 123/12). Peripheral blood from healthy donors was collected and placed in tubes containing sodium citrate. The tubes were then centrifuged at 700× *g* for 10 min, and the blood plasma and leukocyte layer were discarded. The erythrocytes were washed three times with 0.9% sodium chloride (NaCl) solution, and then, a 10% erythrocyte suspension in 0.9% NaCl solution was prepared for the inhibition assays of oxidative hemolysis and malondialdehyde, determined using the method described by Campos et al. [62].

##### Inhibition of Oxidative Hemolysis

The protective effect of HEGP was evaluated according to the method described by Campos et al. [55]. Erythrocytes were pre-incubated at 37 °C for 30 min in the presence of different concentrations of HEGP (5–125 μg/mL). After this period, the samples received only 0.9% NaCl solution to investigate the hemolytic action of the extract or 50 mM AAPH to evaluate its anti-hemolytic activity. As a negative control, erythrocytes were incubated with ethanol at a final concentration of 1%, and ascorbic acid was used as a positive control at the same concentrations as HEGP. The treatments were incubated at 37 °C with periodic homogenization. The hemolysis content was determined after 120, 180 and 240 min of incubation. Samples were centrifuged at 400× *g*/10 min, and the supernatant was transferred to tubes containing 0.9% NaCl solution to perform the reading at 540 nm in a T 70 UV/VIS spectrophotometer (PG Instruments Limited, Leicestershire, UK). As a control of total hemolysis, human erythrocytes were incubated with distilled water. Three independent experiments were performed in duplicate. The percentage of hemolysis was calculated according to the following equation:

Hemolysis (%) = (Abs_sample_/Abs_total hemolysis_) × 100 (3)

##### Dosage of Malondialdehyde (MDA)

The ability of HEGP to inhibit the lipid peroxidation process was investigated in human erythrocytes incubated with the oxidizing agent AAPH, and the MDA concentration was determined as described by Campos et al. [55]. For this experiment, the erythrocytes were pre-incubated at 37 °C for 30 min with different concentrations of HEGP (5–125 μg/mL). Erythrocytes incubated with ethanol at the final concentration of 1% were used as a negative control, and ascorbic acid was used as a positive control at the same concentrations as HEGP. Then, AAPH solution (50 mM) was added and the samples incubated at 37 °C for 4 hours with periodic homogenization. After this time, the samples were centrifuged at 700× *g*/10 min, and 0.5 mL of the supernatant was mixed with 1 mL of 10 nmol thiobarbituric acid (TBA) dissolved in 75 mM potassium phosphate monobasic buffer at pH 2.5. As a standard control, 0.5 mL of MDA solution (20 μM) was incubated with 1 mL of TBA. Samples were maintained at 96 °C for 45 min. After cooling the solution, 4 mL of *n*-butyl alcohol was added, followed by centrifugation at 1600× *g*/5 min. The supernatant from the samples was analyzed at 532 nm in a T 70 UV/VIS spectrophotometer (PG Instruments Limited, Leicestershire, UK). Two independent experiments were performed in duplicate. The MDA levels of the samples were expressed in nmol/mL, obtained by the following formula:

(4) $$\mathrm{MDA}\;\left(\mathrm{nmol}/\mathrm{mL}\right)=\;\mathrm{ABSsample}\;\times \left(\frac{20\times 220.32\;}{\mathrm{ABSstandardMDA}}\right)$$

### 4.5. Anti-Inflammatory Activity—Hyaluronidase Assay

Inhibition of the activity of the hyaluronidase enzyme was determined using the method described by Silva et al. [63] The reaction mixture consisted of 50 μL of HEGP (0.2–75 mg/mL) and 50 μL of the enzyme hyaluronidase (350 units) (type IV-S: bovine tests, Sigma, St. Louis, MO, USA) incubated at 37 °C for 20 min. Then, 1.2 μL of calcium chloride solution (2.5 × 10³ M) was added to activate the enzyme and kept at 37 °C for 20 min. After this period, 0.5 mL of hyaluronic acid sodium salt substrate (0.1 M) was added and the solution incubated at 37 °C for 40 min. Subsequently, 0.1 mL of potassium tetraborate (0.8 M) was added and the solution incubated at 100 °C for 3 min. The solution was then cooled, and 3 mL of *p*-dimethylaminobenzaldehyde was added, followed by incubation at 37 °C for 20 min. Finally, the absorbance was measured at 585 nm in a T 70 UV/VIS spectrophotometer (PG Instruments Limited, Leicestershire, UK), using distilled water as a control. The experiments were performed in triplicate. The percentage of hyaluronidase enzyme inhibition was calculated according to the following equation:

Inhibition of the enzyme hyaluronidase (%) = ((Abs_control_ − Abs_sample_)/Abs_control_) × 100 (5)

### 4.6. Antimutagenic Activity

To investigate the antimutagenic potential of HEGP, strains of *Saccharomyces cerevisiae* D7 diploid (American Type Culture Collection, ATCC 201137) were used, according to the method described by Pascoal et al. [43]. The *S. cerevisiae* colonies were evaluated for the frequency of spontaneous gene conversions at the tryptophan locus and reverse mutations at the isoleucine locus, before experimental use. Culture cells with low spontaneous gene conversion and low reverse mutation frequency were cultured in liquid medium at 28 °C until they reached the stationary growth stage. They then underwent sedimentation and were resuspended in sterile potassium phosphate buffer (0.1 M), pH 7.4, until a final value of 2 × 10^8^ cells/mL was obtained. The mutagenic agent ethyl methanesulfonate (EMS) (5 mg/mL) was added together with HEGP, evaluated at concentrations of 1.5 and 3.0 mg/mL. The mixture was incubated with stirring for 2 h at 37 °C. After this period, the cells were plated in complete and selective medium to determine yeast survival, tryptophan converters and isoleucine reversers. The experiments were performed in triplicate.

### 4.7. Antimicrobial Activity

The antimicrobial activity of HEGP was investigated in microorganisms collected from biological fluids at the Hospital Center and identified in the Microbiology Laboratory of Escola Superior Agrária (ESA) de Bragança, Portugal. Reference strains were obtained from the authorized ATCC distributor (LGC Standards SLU, Barcelona, Spain). The microorganisms used were *Staphylococcus aureus* ATCC 6538™, methicillin-resistant *S. aureus* ESA 175, methicillin-resistant *S. aureus* ESA 159, *Enterococcus faecalis* ATCC 43300™, vancomycin-resistant *E. faecalis* ESA 201, vancomycin-resistant *E. faecalis* ESA 361, *Escherichia coli* ATCC 29998™, cephalosporin-resistant *E. coli* ESA 37, cephalosporin-resistant *E. coli* ESA 54, *Pseudomonas aeruginosa* ATCC 15442, imipenem-resistant *P. aeruginosa* ESA 22, imipenem-resistant *P. aeruginosa* ESA 23, *Cryptococcus neoformans* ATCC 32264, amphotericin B-resistant *C. neoformans* ESA 211, amphotericin B-resistant *C. neoformans* ESA 105, *Candida albicans* ATCC 10231™, amphotericin B-resistant *C. albicans* ESA 100 and amphotericin B-resistant *C. albicans* ESA 97.

The microorganisms were stored in Muller-Hinton medium supplemented with 20% glycerol at 70 °C prior to experimental use. The inoculum was then prepared by dilution of the cell mass in 0.85% NaCl solution, adjusted to 0.5 on the MacFarland scale, as confirmed by spectrophotometric reading at 580 nm for bacteria and 640 nm for yeast. Antimicrobial assays were performed as described by Silva et al. [63] using Nutrient Broth (NB) for bacteria or Yeasts Peptone Dextrose (YPD) for yeast in microplates (96 wells). The HEGP was diluted in dimethylsulfoxide (DMSO) and transferred to the first well, followed by serial dilution. The inoculum was added to all wells (10^4^ Colony Forming Units (CFU)/mL), and the plates were incubated at 37 °C for 24 h for bacteria and at 25 °C for 48 h for yeast. Gentamicin and amphotericin B were used as antibacterial and antifungal controls, respectively. A negative control (medium only), a positive control (inoculated medium) and a DMSO control (DMSO with inoculated medium) were performed in each experiment. After the incubation period, the antimicrobial activity was detected by the addition of 20 μL of 2,3,5-triphenyl-2*H*-tetrazoliumchloride (TTC) solution (5 mg/mL). The minimum inhibitory concentration (MIC) was defined as the lowest concentration of HEGP that visibly inhibited the growth of microorganisms, as indicated by TTC staining, which marks viable cells.

To determine the minimum bactericidal concentration (MBC) and minimum fungicidal concentration (MFC), 20 μL of the last well where growth was observed and from each well where no color changes were seen was seeded in NB or YPD and incubated for 24 h at 37 °C for bacteria growth and 48 h for yeast growth. The lowest concentration that did not result in growth (<10 CFU/plate) after this subculture process was considered the MBC or MFC. The experiments were performed in triplicate, and the results were expressed in mg/mL.

### 4.8. Statistical Analyses

The data are shown as the mean ± SEM and were analyzed for statistically significant differences between groups using one-way analysis of variance (ANOVA) followed by Dunnett’s test for the comparison of more than two groups using the Prism 5 GraphPad Software (GraphPad Software Inc., San Diego, CA, USA). The results were considered significant when *p* < 0.05.

## 5. Conclusions

In conclusion, this study demonstrates the presence of bioactive compounds in the hydroethanolic extract of *M. orbignyi* geopropolis, as well as its antioxidant, anti-inflammatory, antimutagenic and antimicrobial activities.

## Figures and Tables

**Table 1:** Compounds identified from *Melipona orbignyi* geopropolis extract by high performance liquid chromatography coupled to diode array detector and mass spectrometry (HPLC-DAD-MS).

Peak	Identification	Molecular Formula	RT (min)	UV (nm)	[M-H]^−^ *m*/*z*	Error (ppm)	MS/MS *m/z*
**1**	*O*-coumaroyl *O*-galloyl-hexoside	C_22_H_22_O_12_	24.1	289 and 309	477.1029	2.0	313 (C_13_H_13_O_9_)^−^, 271 (C_11_H_11_O_8_)^−^, 169 (C_7_H_5_O_5_)^−^
**2**	*O*-coumaroyl *O*-galloyl-hexoside	C_22_H_22_O_12_	24.8	289 and 309	477.1022	3.4	313 (C_13_H_13_O_9_)^−^, 265 (C_13_H_13_O_6_)^−^, 235 (C_12_H_11_O_5_)^−^, 205 (C_11_H_9_O_4_)^−^, 169 (C_7_H_5_O_5_)^−^
**3**	Aromadendrin	C_15_H_12_O_6_	25.2	289	287.0553	2.7	259 (C_14_H_11_O_5_)^−^, 177 (C_10_H_9_O_3_)^−^
**4**	Di-*O*-galloyl *O*coumaroyl-hexoside	C_29_H_26_O_16_	26.1	286 and 308	629.1122	4.2	465 (C_20_H_17_O_13_)^−^, 459 (C_22_H_19_O_11_)^−^, 313 (C_13_H_13_O_9_)^−^, 271 (C_11_H_11_O_8_)^−^, 169 (C_7_H_5_O_5_)^−^
**5**	*O*-cinnamoyl *O*-galloyl-hexoside	C_22_H_22_O_11_	30.8	280	461.1085	1.0	211 (C_9_H_7_O_6_)^−^, 169 (C_7_H_5_O_5_)^−^, 161 (C_10_H_9_O_2_)^−^
**6**	Di-*O*-galloyl *O*-cinnamoyl-hexoside	C_29_H_26_O_15_	32	280	613.1186	2.1	465 (C_20_H_17_O_13_)^−^, 313 (C_13_H_13_O_9_)^−^, 271 (C_11_H_11_O_8_)^−^, 211 (C_9_H_7_O_6_)^−^, 169 (C_7_H_5_O_5_)^−^
**7**	Di-*O*-coumaroyl-hexoside	C_24_H_24_O_10_	32.4	299 and 311	471.1287	2.0	325 (C_15_H_17_O_8_)^−^, 307 (C_15_H_15_O_7_)^−^, 265 (C_13_H_13_O_6_)^−^, 163 (C_9_H_7_O_3_)^−^, 145 (C_9_H_5_O_2_)^−^
**8**	Naringenin	C_15_H_12_O_5_	32.9	284	271.0614	0.9	–
**9**	Di-*O*-coumaroyl *O*-galloyl-hexoside	C_31_H_28_O_14_	34.3	290 and 311	623.1405	0.2	459 (C_22_H_19_O_11_)^−^, 313 (C_13_H_13_O_9_)^−^, 271 (C_11_H_11_O_8_)^−^, 211 (C_9_H_7_O_6_)^−^, 169 (C_7_H_5_O_5_)^−^,163 (C_9_H_7_O_3_)^−^
**10**	Methyl aromadendrin	C_16_H_14_O_6_	35.3	290	301.0721	1.0	273 (C_15_H_13_O_5_)^−^, 240 (C_14_H_8_O_4_)^−^, 179 (C_8_H_3_O_5_)^−^, 165 (C_8_H_5_O_4_)^−^
**11**	*O*-Cinnamoyl-*O*-coumaroyl-hexoside	C_24_H_24_O_9_	38	285 and 310	455.1359	2.5	163 (C_9_H_7_O_3_)^−^, 145 (C_9_H_5_O_2_)^−^
**12**	*O*-Cinnamoyl *O*-coumaroyl *O*-Galloyl-hexoside	C_31_H_28_O_13_	38.8	285 and 310	607.1462	0.8	461 (C_22_H_21_O_11_)^−^, 443 (C_22_H_19_O_10_)^−^, 313 (C_13_H_13_O_9_)^−^, 271 (C_11_H_11_O_8_)^−^, 211 (C_9_H_7_O_6_)^−^, 169 (C_7_H_5_O_5_)^−^
**13**	Methyl naringenin	C_16_H_14_O_5_	40.3	285	285.0777	2.9	165 (C_8_H_5_O_4_)^−^
**14**	Unknown	C_24_H_22_O_7_	40.9	293	421.1289	1.0	393 (C_23_H_21_O_6_)^−^
**15**	Unknown	C_24_H_22_O_7_	42.1	295	421.1288	0.9	393 (C_23_H_21_O_6_)^−^
**16**	Unknown	C_24_H_22_O_6_	45.3	288	405.1337	1.6	–
**17**	Diterpene	C_20_H_32_O_3_	45.8	–	319.2260	3.4	–
**18**	Diterpene	C_20_H_32_O_3_	46.3	–	319.2261	0.9	–
**19**	Sesquiterpene	C_15_H_22_O_4_	46.9	–	265.1454	3.3	–
**20**	Diterpene ester	C_22_H_34_O_4_	51.9	280	361.2356	4.0	301 (C_20_H_29_O_2_)^−^
**21**	Triterpene	C_30_H_48_O_4_	54	–	471.3459	4.4	–
**22**	Triterpene	C_30_H_48_O_4_	55.3	–	471.3464	3.3	–
**23**	Triterpene	C_30_H_46_O_4_	55.6	–	469.3305	3.9	–
**24**	Unknown	C_24_H_38_O_3_	58.8	–	373.2736	3.3	329 (C_23_H_37_O)^−^
**25**	Triterpene	C_31_H_48_O_3_	62.7	–	467.3509	4.6	–
**26**	Unknown	C_24_H_36_O_3_	64	–	371.2580	3.0	327 (C_23_H_35_O)^−^

RT: retention time; –: non observed/detected means.

**Table 2:** IC_50_ and maximum activity of reference antioxidants and treatments with HEGP.

DPPH^•^				ABTS^•+^		
Samples	IC_50_ (µg/mL)	Maximum Activity		IC_50_ (µg/mL)	Maximum Activity	
		%	µg/mL		%	µg/mL
**Ascorbic acid**	3.2 ± 0.9	93 ± 2.1	10	1.8 ± 0.05	96 ± 2.4	5
**BHT**	20.3 ± 5.6	79 ± 2.6	50	8.1 ± 0.7	98 ± 0.2	50
**HEGP**	18.3 ± 2.8	95 ± 0.5	50	10.3 ± 0.5	98 ± 0.1	50

Values are means ± standard error of the mean (SEM).

**Table 3:** Effect of the hydroethanolic extract of geopropolis of *Melipona orbignyi* (HEGP) on the survival percentage of yeast cells *Saccharomyces cerevisiae* (diploid line D7 ATCC 201137), conversion of genes and mutant colonies.

Treatments		Survival (%)	Gene Conversion Colonies/10^5^	Mutant Colonies/10^6^
HEGP (mg/mL)	EMS (mg/mL)			
0.0	0.0	96.2 ± 2.35	0.77 ± 0.02	0.49 ± 0.01
0.0	5.0	85.2 ± 2.40 ^a^	51.3 ± 0.44 ^a^	391.3 ± 7.80 ^a^
1.5	5.0	49.8 ± 1.11 ^b^	43.8 ± 2.79 ^b^	73.7 ± 4.35 ^b^
3.0	5.0	38.1 ± 3.79 ^c^	37.2 ± 1.32 ^b^	53.2± 1.71 ^b^

a, b, c: Means with different superscripts are significantly different for each attribute (*p* < 0.05). Values are means ± SEM. EMS, Ethyl methanesulfonate.

**Table 4:** Minimum inhibitory concentration (MIC), minimum bactericidal concentration (MBC), and minimum fungicidal concentration (MFC) for HEGP from *M. orbignyi*.

**Microorganisms**	**HEGP (mg/mL)**		**Gentamicin (µg/mL)**
**Gram-positive bacteria**	**MIC**	**MBC**	**MBC**
*Staphylococcus aureus* ATCC 6538™	6.13 ± 0.10	8.50 ± 0.28	2.00 ± 0.28
*S. aureus* ESA 175 Methicillin-resistant	6,42 ± 0.46	8.75 ± 0.62	2.67 ± 0.16
*S. aureus* ESA 159 Methicillin-resistant	6.92 ± 0.30	9.08 ± 0.58	2.50 ± 0.28
*Enterococcus faecalis* ATCC 43300™	7.08 ± 0.58	10.5 ± 0.22	2.83 ± 0.30
*E. faecalis* ESA 201 Vancomycin-resistant	8.17 ± 0.44	10.9 ± 0.30	3.25 ± 0.14
*E. faecalis* ESA 361 Vancomycin-resistant	9.08 ± 0.08	11.2 ± 0.38	3.33 ± 0.16
**Gram-negative bacteria**			
*Escherichia coli* ATCC 29998™	10.5 ± 0.82	13.2 ± 0.52	4.58 ± 0.30
*E. coli* ESA 37 Cephalosporins-resistant	11.2 ± 0.32	13.8 ± 0.72	4.67 ± 0.22
*E. coli* ESA 54 Cephalosporins-resistant	11.5 ± 0.76	13.7 ± 0.90	4.92 ± 0.08
*Pseudomonas aeruginosa* ATCC 15442™	12.9 ± 0.50	16.5 ± 0.30	5.00 ± 0.28
*P.aeruginosa* ESA 22 Imipenem-resistant	13.3 ± 0,60	17.0 ± 1.08	6.17 ± 0.16
*P.aeruginosa* ESA 23 Imipenem-resistant	13.5 ± 1.22	17.7 ± 1.02	6.50 ± 0.28
**Microorganisms**	**HEGP (mg/mL)**		**Amphotericin-B (µg/mL)**
**Fungi**	**MIC**	**MFC**	**MFC**
*Cryptococcus neoformans* ATCC 32264 ™	19.3 ± 0.60	25.0 ± 1.04	0.13 ± 0.07
*C. neoformans* ESA 211 Amphotericin-B resistant	20.9 ± 0.79	26.0 ± 1.04	0.25 ± 0.14
*C. neoformans* ESA 105 Amphotericin-B resistant	21.4 ± 1.10	26.1 ± 1.58	0.38 ± 0.22
*Candida albicans* ATCC 10231™	23.0 ± 1.06	34.0 ± 1.12	0.29 ± 0.16
*C.albicans* ESA 100 Amphotericin-B resistant	23.7 ± 1.29	35.0 ± 1.23	0.14 ± 0.08
*C.albicans* ESA 97 Amphotericin-B resistant	24.4 ± 1.83	36.1 ± 0.50	0.25 ± 0.14

Values are means ± SEM.

## Abbreviations

AAPH	2,2′-Azobis (2-methylpropionamidine) dihydrochloride
ABTS^•+^	2,2′-Azino-bis(3-ethylbenzothiazoline-6-sulphonic acid)
ATCC	American Type Culture Collection
BHT	Butylated hydroxytoluene
DPPH^•^	2,2-Diphenyl-1-picrylhydrazyl
EMS	Ethyl methanesulfonate
ESA	Escola Superior Agrária
GAE	Gallic acid equivalents
HEGP	Hydroalcoholic extract of geopropolis
MDA	Malondialdehyde
MBC	Minimum bactericidal concentration
MIC	Minimum inhibitory concentration
MFC	Minimum fungicidal concentration
*M. orbignyi*	*Melipona orbignyi*
NaCl	Sodium chloride
QE	Quercetin equivalents
SEM	Standard error of the mean
TBA	Thiobarbituric acid
TTC	2,3,5-Triphenyl-2*H*-Tetrazoliumchloride

## References

[1] Freitas M.O., Ponte F.A., Lima M.A.S., Silveira E.R. (2008). Flavonoids and triterpenes from the nest of the stingless bee *Trigona spinipes*. J. Braz. Chem. Soc..

[2] Bartolomeu A.R., Frión-Herrera Y., da Silva L.M., Romagnoli G.G., de Oliveira D.E., Sforcin J.M. (2016). Combinatorial effects of geopropolis produced by *Melipona fasciculata* Smith with anticancer drugs against human laryngeal epidermoid carcinoma (HEp-2) cells. Biomed. Pharmacother..

[3] Souza R.C., Paiva L.K.O.Y.J., Aguiar L., Plácido F., Oliveira M. (2004). Valor nutricional do mel e pólen de abelhas sem ferrão da região amazônica. Acta Amazon..

[4] Araújo M.J.A.M., Búfalo M.C., Conti B., Junior A.F., Trusheva B., Bankova V., Sforcin J.M. (2015). The chemical composition and pharmacological activities of geopropolis produced by *Melipona fasciculata* Smith in Northeast Brazil. J. Mol. Pathophysiol..

[5] Cunha M.G., Franchin M., Galvão L.C.D.C., Bueno-Silva B., Ikegaki M., Alencar S.M., Rosalen P.L. (2013). Apolar bioactive fraction of *Melipona scutellaris* geopropolis on *Streptococcus mutans* biofilm. Evid.-Based Complement. Altern. Med..

[6] Franchin M., Cunha M.G., Denny C., Napimoga M.H., Cunha T.M., Bueno-Silva B., Alencar S.M., Ikegaki M., Rosalen P.L. (2013). Bioactive fraction of geopropolis from *Melipona scutellaris* decreases neutrophils migration in the inflammatory process: Involvement of nitric oxide pathway. Evid.-Based Complement. Altern. Med..

[7] Dutra R.P., Abreu B.V.B., Cunha M.S., Batista M.C.A., Torres L.M.B., Nascimento F.R.F., Ribeiro M.N.S., Guerra R.N.M. (2014). Phenolic acids, hydrolyzable tannins, and antioxidant activity of geopropolis from the stingless bee *Melipona fasciculata* Smith. J. Agric. Food Chem..

[8] Moure’s bee catalogue. http://moure.cria.org.br.

[9] Nogueira-Neto P. (1997). Arquitetura dos ninhos. Vida e criação de abelhas indígenas sem ferrão.

[10] Souza S.A., Camara C.A., Silva E.M.S., Silva T.M.S. (2013). Composition and antioxidant activity of geopropolis collected by *Melipona subnitida* (Jandaíra) bees. Evid.-Based Complement. Altern. Med..

[11] Franchin M., Cunha M.G., Denny C., Napimoga M.H., Cunha T.M., Koo H., Alencar S.M., Ikegaki M., Rosalen P.L. (2012). Geopropolis from *Melipona scutellaris* decreases the mechanical inflammatory hypernociception by inhibiting the production of IL-1β and TNF-α. J. Ethnopharmacol..

[12] Ribeiro-Junior J.A., Franchin M., Cavallini M.E., Denny C., Alencar S.M., Ikegaki M., Rosalen P.L. (2015). Gastroprotective effect of geopropolis from *Melipona scutellaris* is dependent on production of nitric oxide and prostaglandin. Evid.-Based Complement. Altern. Med..

[13] Coelho G.R., Mendonça R.Z., Vilar K.D.S., Figueiredo C.A., Badari J.C., Taniwaki N., Namiyama G., Oliveira M.I., Curti S.P., Silva P.E. (2015). Antiviral action of hydromethanolic extract of geopropolis from *Scaptotrigona postica* against antiherpes simplex virus (HSV-1). Evid.-Based Complement. Altern. Med..

[14] Dutra R.P., Nogueira A.M.C., Marques R.R.D.O., Costa M.C.P., Ribeiro M.N.S. (2008). Pharmacognostic evaluation of geopropolis of *Melipona fasciculata* Smith from Baixada Maranhense, Brazil. Rev. Bras. Farmacogn..

[15] Chemical Abstract Service database. https://scifinder.cas.org.

[16] Campos J.F., Santos U.P., Macorini L.F.B., Melo A.M.M.F., Balestieri J.B.P., Gamero E.J.P., Cardoso C.A.L., Souza K.P., Santos E.L. (2014). Antimicrobial, antioxidant and cytotoxic activities of propolis from *Melipona orbignyi* (Hymenoptera, Apidae). Food Chem. Toxicol..

[17] Markham K.R. (1982). Ultraviolet-visible absorption spectroscopy. Techniques of Flavonoid Identification.

[18] Cuyckens F., Claeys M. (2004). Mass spectrometry in the structural analysis of flavonoids. J. Mass Spectrom..

[19] Justino G.C., Borges C.M., Florencio M.H. (2009). Electrospray ionization tandem mass spectrometry fragmentation of protonated flavone and flavonol aglycones: A re-examination. Rapid. Commun. Mass Spectrom..

[20] Bankova V., Christov R., Marcucci C., Popov S. (1998). Constituents of Brazilian geopropolis. Z. Naturforsch. C.

[21] Costa da Silva E.C., Muniz M.P., Nunomura R.C.S., Nunomura S.M., Zilse G.A.C. (2013). Phenolic constituents and antioxidant activity of geopropolis from two species of Amazonian stingless bees. Quim. Nov..

[22] Wang J.B., Qin Y., Kong W.J., Wang Z.W., Zeng L.N., Fang F., Jin C., Zhao Y.L., Xiao X.H. (2011). Identification of the antidiarrhoeal components in official rhubarb using liquid chromatography–tandem mass spectrometry. Food Chem..

[23] Walker P., Crane E. (1987). Constituents of propolis. Apidologie.

[24] Patricio E.F.L.R.A., Cruz-Lopez L., Maile R., Tentschert J., Jones G.R., Morgan E.D. (2002). The propolis of stingless bees: Terpenes from the tibia of three *Frieseomelitta* species. J. Insect Physiol..

[25] Marquez Hernandez I., Cuesta-Rubio O., Campo Fernandez M., Perez A.R., Porto R.M.O., Piccinelli A.L., Rastrelli L. (2010). Studies on the constituents of yellow cuban propolis: GC-MS determination of triterpenoids and flavonoids. J. Agric. Food Chem..

[26] Cunha M.G., Rosalen P.L., Franchin M., Alencar S.M., Ikegaki M., Ransom T., Beutler J.A. (2016). Antiproliferative constituents of geopropolis from the bee *Melipona scutellaris*. Planta Med..

[27] Carocho M., Ferreira I.C. (2013). A review on antioxidants, prooxidants and related controversy: Natural and synthetic compounds, screening and analysis methodologies and future perspectives. Food Chem. Toxicol..

[28] Kumar S., Pandey A.K. (2013). Chemistry and biological activities of flavonoids: An overview. Sci. World J..

[29] Wang W., Zeng S.F., Yang C.R., Zhang Y.J. (2009). A new hydrolyzable tannin from *Balanophora harlandii* with radical-scavenging activity by scavenging activity. Helv. Chim. Acta.

[30] Karamać M. (2009). Chelation of Cu (II), Zn (II), and Fe (II) by tannin constituents of selected edible nuts. Int. J. Mol. Sci..

[31] Figueroa-Espinoza M.C., Zafimahova A., Alvarado P.G.M., Dubreucq E., Poncet-Legrand C. (2015). Grape seed and apple tannins: Emulsifying and antioxidant properties. Food Chem..

[32] Ramachandran S., Prasad N.R. (2008). Effect of ursolic acid, a triterpenoid antioxidant, on ultraviolet-B radiation-induced cytotoxicity, lipid peroxidation and DNA damage in human lymphocytes. Chem. Biol. Interact..

[33] Ramachandran S., Prasad R., Pugalendi K.V., Menon V.P. (2008). Modulation of UVB-induced oxidative stress by ursolic acid in human blood lymphocytes. Asian J. Biochem..

[34] Shoaib M., Shah I., Ali N., Adhikari A., Tahir M.N., Shah S.W., Ishtiaq S., Khan J., Khan S., Umer M.N. (2017). Sesquiterpene lactone! A promising antioxidant, anticancer and moderate antinociceptive agent from *Artemisia macrocephala* jacquem. BMC Complement. Altern. Med..

[35] Valko M., Rhodes C.J., Moncol J., Izakovic M., Mazur M. (2006). Free radicals, metals and antioxidants in oxidative stress-induced cancer. Chem. Biol. Interact..

[36] Lobo V., Patil A., Phatak A., Chandra N. (2010). Free radicals, antioxidants and functional foods: Impact on human health. Pharmacogn. Rev..

[37] Reuter S., Gupta S.C., Chaturvedi M.M., Aggarwal B.B. (2010). Oxidative stress, inflammation, and cancer: How are they linked?. Free Radic. Biol. Med..

[38] Mackay C.R. (2008). Moving targets: Cell migration inhibitors as new anti-inflammatory therapies. Nat. Immunol..

[39] Rawdin B.J., Mellon S.H., Dhabhar F.S., Epel E.S., Puterman E., Su Y., Burke H.M., Reus V.I., Rosser R., Hamilton S.P. (2013). Dysregulated relationship of inflammation and oxidative stress in major depression. Brain Behav. Immun..

[40] Agil A., Reiter R.J., Jiménez-Aranda A., Ibán-Arias R., Navarro-Alarcón M., Marchal J.A., Adem A., Fernández-Vázquez G. (2013). Melatonin ameliorates low-grade inflammation and oxidative stress in young Zucker diabetic fatty rats. J. Pineal Res..

[41] Fernández-Sánchez A., Madrigal-Santillán E., Bautista M., Esquivel-Soto J., Morales-González Á., Esquivel-Chirino C., Durante-Montiel I., Sánchez-Rivera G., Valadez-Veja C., Morales-González J.A. (2011). Inflammation, oxidative stress, and obesity. Int. J. Mol. Sci..

[42] Kogan G., Šoltés L., Stern R., Gemeiner P. (2007). Hyaluronic acid: A natural biopolymer with a broad range of biomedical and industrial applications. Biotechnol. Lett..

[43] Pascoal A., Rodrigues S., Teixeira A., Feás X., Estevinho L.M. (2014). Biological activities of commercial bee pollens: Antimicrobial, antimutagenic, antioxidant and anti-inflammatory. Food Chem. Toxicol..

[44] Formisano C., Sanna C., Ballero M., Chianese G., Sirignano C., Rigano D., Millán E., Muñoz E., Taglialatela-Scafati O. (2017). Anti-inflammatory sesquiterpene lactones from *Onopordum illyricum* L. (Asteraceae), an Italian medicinal plant. Fitoterapia.

[45] Terra X., Montagut G., Bustos M., Llopiz N., Ardèvol A., Bladé C., Fernández-Larrea J., Pujadas G., Salvadó J., Arola L. (2009). Grape-seed procyanidins prevent low-grade inflammation by modulating cytokine expression in rats fed a high-fat diet. J. Nutr. Biochem..

[46] Soobrattee M.A., Neergheen V.S., Luximon-Ramma A., Aruoma O.I., Bahorun T. (2005). Phenolics as potential antioxidant therapeutic agents: Mechanism and actions. Mutat. Res..

[47] Costa G., Francisco V., Lopes M.C., Cruz M.T., Batista M.T. (2012). Intracellular signaling pathways modulated by phenolic compounds: Application for new anti-inflammatory drugs discovery. Curr. Med. Chem..

[48] Venditti A., Serrilli A.M., Rizza L., Frasca G., Cardile V., Bonina F.P., Bianco A. (2012). Aromadendrine, a new component of the flavonoid pattern of *Olea europaea* L. and its anti-inflammatory activity. Nat. Prod. Res..

[49] Simões-Ambrosio L.M., Gregório L.E., Sousa J.P., Figueiredo-Rinhel A.S., Azzolini A.E., Bastos J.K., Lucisano-Valim Y.M. (2010). The role of seasonality on the inhibitory effect of Brazilian green propolis on the oxidative metabolism of neutrophils. Fitoterapia.

[50] Choudhari S.K., Chaudhary M., Gadbail A.R., Sharma A., Tekade S. (2014). Oxidative and antioxidative mechanisms in oral cancer and precancer: A review. Oral Oncol..

[51] Cano-Lamadrid M., Marhuenda-Egea F.C., Hernández F., Rosas-Burgos E.C., Burgos-Hernández A., Carbonell-Barrachina A.A. (2016). Biological activity of conventional and organic pomegranate juices: Antioxidant and antimutagenic potential. Plant Foods Hum. Nutr..

[52] Fernandes F.H., Guterres Z.R., Garcez W.S., Lopes S.M., Corsino J., Garcez F.R. (2014). Assessment of the (anti) genotoxicity of brown propolis extracts from Brazilian Cerrado biome in a *Drosophila melanogaster* model. Food Res. Int..

[53] Cai Y., Luo Q., Sun M., Corke H. (2004). Antioxidant activity and phenolic compounds of 112 traditional Chinese medicinal plants associated with anticancer. Life Sci..

[54] Islam M.T., Mata A.M.O.F., Aguiar R.P.S., Paz M.F.C.J., Alencar M.V.O.B., Ferreira P.M.P., Melo-Cavalcante A.A.C. (2016). Therapeutic potential of essential oils focusing on diterpenes. Phytother. Res..

[55] Campos J.F., Santos U.P., Rocha P.S., Damião M.J., Balestieri J.B.P., Cardoso C.A.L., Gamero E.J.P., Estevinho L.M., Picoli Souza K., Santos E.L. (2015). Antimicrobial, antioxidant, anti-inflammatory, and cytotoxic activities of propolis from the stingless bee *Tetragonisca fiebrigi* (Jataí). Evid.-Based Complement. Altern. Med..

[56] Borges A., Ferreira C., Saavedra M.J., Simoes M. (2013). Antibacterial activity and mode of action of ferulic and gallic acids against pathogenic bacteria. Microb. Drug Resist..

[57] Laxminarayan R., Duse A., Wattal C., Zaidi A.K., Wertheim H.F., Sumpradit N., Vlieghe E., Hara G.L., Gould I.M., Goossens H. (2013). Antibiotic resistance—The need for global solutions. Lancet Infect. Dis..

[58] Hawkey P.M., Jones A.M. (2009). The changing epidemiology of resistance. J. Antimicrob. Chemother..

[59] Cushnie T.P.T., Lamb A.J. (2005). Antimicrobial activity of flavonoids. Int. J. Antimicrob. Agents.

[60] Vattem D.A., Lin Y.T., Labbe R.G., Shetty K. (2004). Phenolic antioxidant mobilization in cranberry pomace by solid-state bioprocessing using food grade fungus *Lentinus edodes* and effect on antimicrobial activity against select food borne pathogens. Innov. Food Sci. Emerg. Technol..

[61] Ekambaram S.P., Perumal S.S., Balakrishnan A. (2016). Scope of hydrolysable tannins as possible antimicrobial agent. Phytother. Res..

[62] Campos J.F., Castro D.T.H., Damião M.J., Torquato H.F.V., Paredes-Gamero E.J., Carollo C.A., Estevinho L.M., Picoli Souza K., Santos E.L. (2016). The chemical profile of *Senna velutina* leaves and their antioxidant and cytotoxic effects. Oxid. Med. Cell Longev..

[63] Silva J.C., Rodrigues S., Feás X., Estevinho L.M. (2012). Antimicrobial activity, phenolic profile and role in the inflammation of propolis. Food Chem. Toxicol..

